# Insecticidal Effect of Labramin, a Lectin—Like Protein Isolated from Seeds of the Beach Apricot Tree, *Labramia bojeri*, on the Mediterranean Flour Moth, *Ephestia kuehniella*

**DOI:** 10.1673/031.012.6201

**Published:** 2012-05-20

**Authors:** Diego Stéfani Teodoro Martinez, Maria das Graças Machado Freire, Paulo Mazzafera, Roberto Theodoro Araujo-Júnior, Rafael Delmond Bueno, Maria Lígia Rodrigues Macedo

**Affiliations:** ^1^ Laboratório de Purificação de Proteínas e suas Funções Biológicas, Departamento de Tecnologia de Alimentos e Saúde Pública, Centro de Ciências Biológicas e da Saúde, Universidade Federal de Mato Grosso do Sul (UFMS), Campo Grande, MS, Brazil; ^2^ Departamento de Bioquímica, Instituto de Biologia, Universidade Estadual de Campinas (UNICAMP), Campinas, SP, Brazil; ^3^ Laboratório de Química e Biomoléculas (LAQUIBIO), Centro de Pesquisas e Pós-graduação, lnstitutos Superiores do CENSA (ISECENSA), Campos do Goytacazes, RJ, Brazil; ^4^ Departamento de Fisiologia Vegetal, Instituto de Biologia, Universidade Estadual de Campinas (UNICAMP), Campinas, SP, Brazil

**Keywords:** insecticidal protein, *Ephestia kuehniella*, Lepidoptera

## Abstract

The objective of this work was to study the insecticidal effect of labramin, a protein that shows lectin—like properties. Labramin was isolated from seeds of the Beach Apricot tree, *Labramia bojeri* A. DC ex Dubard (Ericales: Sapotaceae), and assessed against the development of the Mediterranean flour moth *Ephestia kuehniella* Zeller (Lepidoptera: Pyralidae), an important pest of stored products such as corn, wheat, rice, and flour. Results showed that labramin caused 90% larval mortality when incorporated in an artificial diet at a level of 1% (w/w). The presence of 0.25% labramin in the diet affected the larval and pupal developmental periods and the percentage of emerging adults. Treatments resulted in elevated levels of trypsin activity in midgut and fecal materials, indicating that labramin may have affected enzyme—regulatory mechanisms by perturbing peritrophic membranes in the midgut of is. *kuehniella* larvae. The results of dietary experiments with *E*. *kuehniella* larvae showed a reduced efficiency for the conversion of ingested and digested food, and an increase in approximate digestibility and metabolic cost. These findings suggest that labramin may hold promise as a control agent to engineer crop plants for insect resistance.

## Introduction

The Mediterranean flour moth, *Ephestia kuehniella* Zeller (Lepidoptera: Pyralidae), is widely distributed in tropical and temperate regions of the world. It can be found in a great variety of foodstuffs including flour, grain residues (insect—infected grain, broken kernels, and dust), and various whole grains (Ayvaz et al. 2008). Control of these insects generally requires the use of chemical insecticides, although these insecticides are toxic to humans and domestic animals, and negatively impact the environment (Boobis et al. 2008).

Plants have protective mechanisms that allow them to successfully resist unfavorable conditions, including attack by insects and phytopathogenic microorganisms. A mechanism to enhance the resistance of important crops is the use of plant insecticidal proteins, such as protease inhibitors, arcelins, chitinases, ribosome—inactivating proteins, and lectins (Carlini and Grossi-de-Sá 2002; Hosseininaveh et al. 2009).

The insecticidal activity of carbohydrate—binding plant lectins against insects of the orders Coleoptera, Diptera, Lepidoptera, and Homoptera has been studied (Macedo et al. 2003; Oliveira et al. 2011). The identification of an increasing number of plant lectins showing insecticidal activity towards economically important pest species has fueled growing interest in their potential use in the field for the engineering of crops (Sharma et al. 2004; Fitches et al. 2008). During the last decade, evidence has accumulated to indicate that plants also synthesize lectins in minute amounts in response to some specific stress factors and changing environmental conditions (Vandenborre et al. 2011). Although the precise mode of action of insecticidal plant lectins is not fully understood, these proteins can act as recognition molecules in cell—cell or cell—matrix interactions, and may bind to the PM (peritrophic membrane) and/or chitinous structures in the midgut region of insects; this binding is necessary for lectins to exert their deleterious effects in insects. In addition, lectins are frequently resistant to proteolytic degradation by insect digestive enzymes and assimilatory proteins, and are therefore available to inhibit food digestion and absorption (Macedo et al. 2010).

Macedo et al. (2004a) purified a novel protein from the seeds of the Beach Apricot tree *Labramia bojeri* A. DC ex Dubard (Ericales: Sapotaceae) seeds. Labramin is a homologue of the kunitz—type trypsin inhibitor and has lectin—like properties. The study of the specificity of the lectin—like activity of labramin showed that it has higher affinity for glycoproteins and N-acetylglucosamine. This affinity was verified for insecticidal and physiological proteins, such as Arcelin-1 lectin—like (Fabre et al. 1998) and typical plant lectins (Machuka et al. 1999). The objective of this work was to study the insecticidal effect of labramin against the *E*. *kuehniella*. The effect of labramin was evaluated during different stages of insect development and by analyzing nutritional parameters and the activity of trypsin, the main enzyme for protein digestion in *E*. *kuehniella*.

## Materials and Methods

### Materials

The seeds of *L*. *bojeri* used in this study were collected in the state of Rio de Janeiro, Brazil. N-benzoyl-DL-arginyl-p-nitroanilide (BApNA) was supplied from Sigma-Aldrich (www.sigmaaldrich.com). All other chemicals were of reagent grade and obtained from local suppliers.

### Insect cultures

A culture was originally supplied by Dr. J.R.P. Parra (Laboratory of Insect Biology, University of São Paulo, Piracicaba, São Paulo, Brazil), and was routinely maintained on artificial diet prepared by mixing wheat flour, whole wheat husks, whole wheat, and yeast (8:2:1.9:0.1) in the Laboratory of Protein Purification and its Biological Functions of the Federal University of Mato Grosso do Sul, Brazil.

### Extraction and purification of labramin

Labramin was prepared according to Macedo et al. (2004a). Seeds of *L*. *bojeri* were ground and extracted in 150 mM NaCl buffer (1:5 w:v ratio) at 4 ^°^C for 24 hours, then centrifuged at 10,000 × g (30 min; 4 °C). The clear supernatant (crude extract) was used to determine the protein content and hemagglutinating activity. Pure labramin was obtained in three chromatographic steps: Sephacryl S-400, DEAE-sepharose, and reverse phase HPLC on Cl8 µ-Bondapack column.

Protein concentrations were determined by the dye—binding method of Bradford (1976), with bovine serum albumin as the standard.

### Insect bioassays

Insect bioassays were maintained in plastic boxes (5.0 ×3.0 cm) with perforated plastic covers at 65–75% RH, 16:8 L:D photoperiod, and a temperature of 28 ±1 ^°^C.

To examine the effects of labramin on *E*. *kuehniella*, larvae were reared on an artificial diet containing 0.25, 0.50, or 1.00% (w/w) labramin. An artificial diet without labramin was used as the control. Each treatment was repeated five times using 10 neonate larvae per replicate, and larval weights and survival were recorded in the fourth instar.

A total of 75 individuals were reared from neonate to adult stage on artificial diet containing the control diet or 0.25% (w/w) labramin. The intermolt durations and mortalities were recorded daily. In addition, surviving fourth instars were used to assess the effects of labramin on digestion and the trypsin—like activity of the midgut. Feces were separated and stored at —20 ^°^C until required.

### Measurement of nutritional parameters

Nutritional parameters were compared between fourth instar larvae that were exposed to either a labramin—treated (0.25% w/w) or a control diet. Five *E*. *kuehniella* larvae (newly hatched) were placed in plastic boxes (*n* = 50) containing a known weight of the diet. Fourth instar larvae were examined at 25 days after treatment. Larvae, feces, and the remaining uneaten diet were separated, dried, and weighed. The following nutritional indices were then measured according Scriber and Slansky Jr. (1981):

*Efficiency of conversion of ingested food* (ECI) estimates the percentage of ingested food that is converted to biomass, calculated as: (biomass gained (mg fresh mass) / food ingested (mg dry mass)) ×100.

*Efficiency of conversion of digested food* (ECD) estimates the efficiency with which digested food is converted to biomass, calculated as: biomass gained (mg fresh mass)/(food ingested (mg dry mass) — feces (mg dry mass)) ×100. *Metabolic cost* (MC) was calculated as 100 — ECD.

*Approximate digestibility* (AD) estimates the amount of ingested food that is digested, and was calculated as: (food ingested (mg dry mass) — feces (mg dry mass))/food ingested (mg dry mass) ×100.

### Midgut preparation

Homogenates of the larval guts were prepared according to Macedo et al. (1993). Fourth instar larvae were cold—immobilized and their midguts removed in cold 150 mM NaCl and stored frozen at —20 ^°^C until use. Later the midguts were homogenized in 150 mM NaCl, centrifuged at 6000 × g for five min at 8 ^°^C and the supernatants (fluid midgut) were pooled and kept on ice for enzymatic assays.

### Preparation of fecal samples

The fecal samples of fourth instars after treatment were homogenized and centrifuged as described above, then the supernatants were pooled and kept on ice for enzymatic assays.

### Enzymatic assay

Trypsin—like enzymes were assayed using BApNA as substrate. For routine assays, Nbenzoyl-DL-arginyl-p-nitroanilide (BApNA) was used at a final concentration of 1 mM in 1% (v/v) DMSO. Aliquots (50 µg of protein) of midgut larval extracts and feces were incubated in 50 mM Tris-HCl buffer, pH 8.0, in a final volume of 0.1 mL for 10 min, before adding 1 mL of substrate. The reaction was allowed to proceed at 37 ^°^C for 20 min and then stopped by adding 0.2 mL of 30% (v/v) acetic acid. The resulting absorbance was read at 410 nm. Each assay was carried out in triplicate. The linearity of the relationship between the changes in absorbance with time was checked to ensure substrate concentrations were not limiting. Substrate and enzyme controls were run to ensure the validity of sample absorbance readings. Protein concentrations were determined by the dye—binding method of Bradford (1976), with bovine serum albumin as the standard.

### Digestion of labramin

Larval gut homogenates were prepared as described above. For this, 10 larval midguts of *E*. *kuehniella* (fourth instar) were dissected and extracted in 1 mL of 0.1 M Tris, pH 8.0, and processed as described previously. Labramin was incubated with this homogenate in Tris buffer (final concentration, 2 mg/mL). The labramin/midgut protein ratio was 1:10 (w/w). The digestion was carried out for 0, 6, 12, 24, and 48 hours at 37 ^°^C and was stopped by immersing the tubes in boiling water for two min. The degradation of BSA was used as a positive control for serine protease activity. The proteins were subsequently separated by SDS-PAGE on 12.5% Polyacrylamide gels as described by Laemmli (1970).

### Statistical analysis

All data were examined using the Mann-Whitney Test. Statistical significance was considered at *the p* < 0.05 level.

## Results

### Effect of labramin on fourth—instar larvae

**Figure 1.**
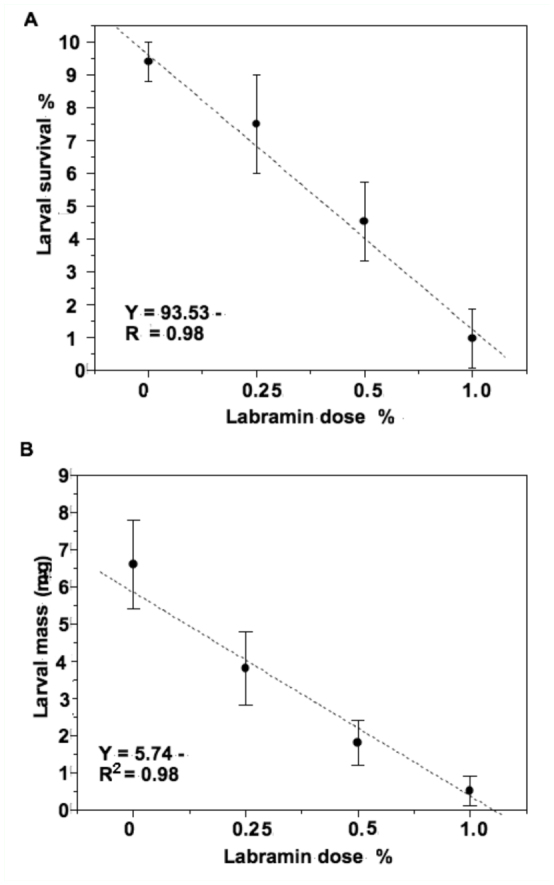
Effect of dietary labramin on *Ephestia kuehniella* artificial larval: (A) Survival (%) and (B) weight (mg) using an artificial diet bioassay. Each point has *n* = 50. Bars indicate SE of the mean. The same letters indicate that there were no significant statistical differences (Mann-Whitney ), U,n= 50, *p <* 0.05). High quality figures are available online.

The effect of labramin on the development of *A*. *kuehniella* was assessed by determining the number and weight of surviving larvae (fourth—instar) fed on a diet containing increasing amounts of labramin (Figure 1). The mortality and weight of larvae fed with the control diet were about 5% and 5.7 mg, respectively, and when fed with 1.0% labramin were about 90% and 0.5 mg, respectively The regression analysis shows that 0.5 and 0.48% labramin caused a 50% reduction in the average survival (LD_50_) and weight (ED_50_) of the larvae, respectively, compared to the control.

### Effects of labramin on developmental stages

The larval and pupal developmental times of larvae fed on labramin diet at 0.25% were significantly longer. The larval period increased by 14 days and the pupal period by two days, resulting in a prolonged period of development from neonate larvae to adults. Labramin treatment caused 57% mortality of *E*. *kuehniella*, while the emergence rate of control larvae was 85%. These results are summarized in Table 1.

### Nutritional parameters

**Figure 2.**
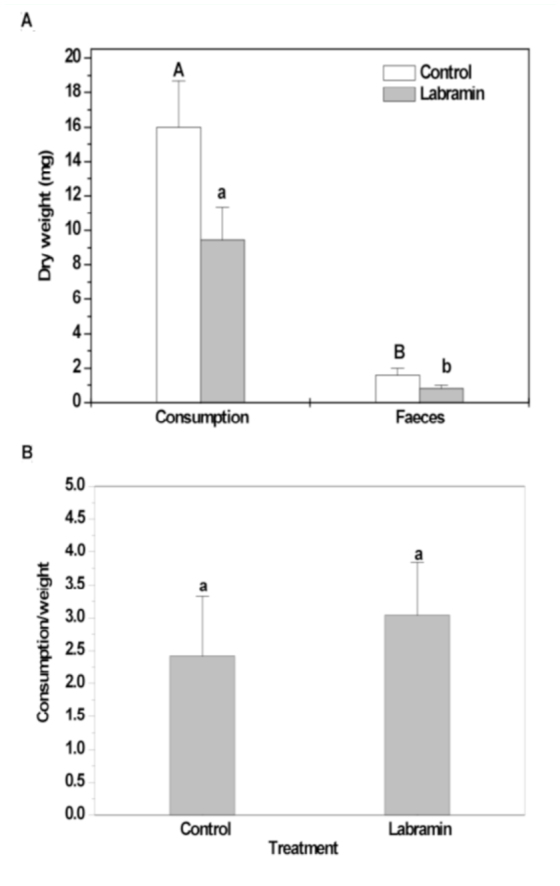
Nutritional parameters measured for *Ephestia kuehniella* larvae in the 0.25% labramin (w/w) feeding trial. (A) Diet consumption and fecal production by larvae of fourth–instar. (B) Mean diet consumption as a ratio to mean larval body weight. Bars indicate SE of the mean. The same letters indicate that there were no significant statistical differences between the control and labramin treatments (Mann-Whitney U, *n* = 50, *p <* 0.05). High quality figures are available online.

Labramin incorporated into artificial diet at 0.25% (w/w) significantly affected the nutritional parameters of *A*. *kuehniella* fourth— instar larvae. Larvae reared on a labramin— containing diet consumed less and produced less feces than the control group. As shown in Figure 2A, the mean diet consumption and fecal production in labramin—fed larvae were reduced by 41% and 50%, respectively, compared to the control. No significant differences were observed in the consumption of the diet when assessed as a ratio of the body weight for both the control and the 0.25% labramin groups (Figure 2B). Labramin reduced the conversion of ingested (ECI) and digested food (ECD) into biomass by *A*. *kuehniella* larvae by approximately 27% and 26%, respectively. Labramin—fed larvae presented a higher metabolic cost (MC) and approximate digestibility (AD) indices of about 32% and 91%, respectively, compared to the control larvae (Table 2).

### Trypsin—like activity

**Figure 3.**
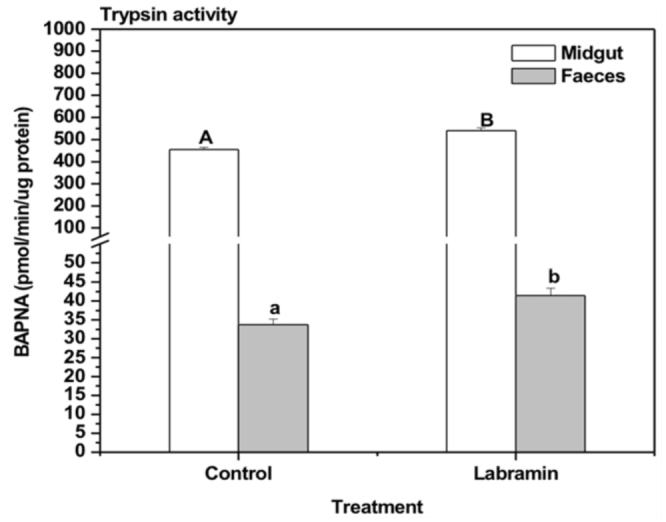
Trypsin activities in midgut and feces from *Ephestia kuehniella* larvae after exposure to 0.25% labramin in the feeding trial. Larvae were fed for four instars (25 days) on control diet or diets containing 0.25% labramin. Enzyme activities are expressed as mean of total pmols of product per minute per µg protein. The product was p-nitroanilide for trypsin assay. Bars indicate SE of the mean. Different letters denote a significant difference between the control and labramin treatments (Mann-Whitney U, *n* = 50, *p* < 0.05). High quality figures are available online.

The labramin diet significantly increased total trypsin activity in the midgut by approximately 19%. Elevated levels of trypsin activity in fecal material were also observed. Labramin—fed larvae had an approximately 23% higher activity compared to that of controls (activity per µg feces protein; Figure 3).

**Table 1.**
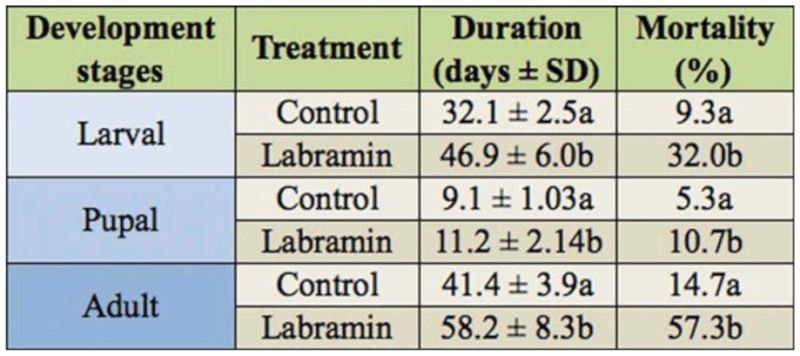
Duration of developmental stages and mortality of *Ephestia kuehniella* fed on an artificial diet containing 0.25% labramin (w/w).

**Table 2.**
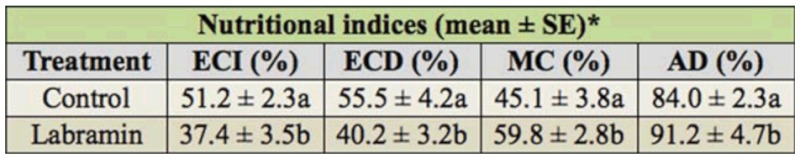
Nutritional indices of *Ephestia kuehniella* fourth instar larvae fed on 0.25% labramin treated artificial diet.

### Digestion of labramin and gel electrophoresis

**Figure 4.**
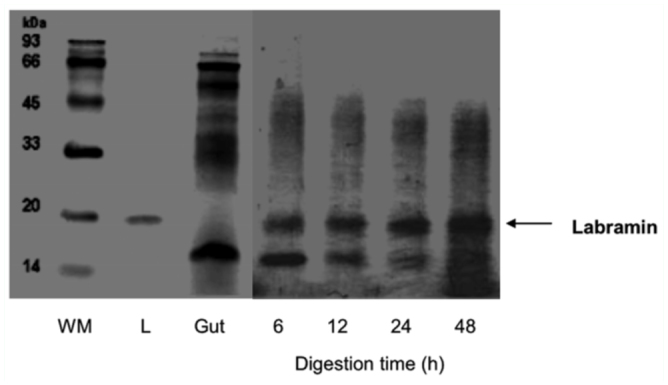
SDS-PAGE of labramin digested by midgut extracts of *Ephestia kuehniella*. High quality figures are available online.

The susceptibility of labramin to degradation by *A*. *kuehniella* midgut proteolytic enzymes was assessed by incubating the lectin with these enzymes followed by SDS-PAGE (Figure 4). Incubation of labramin with *A*. *kuehniella* midgut extracts for up to 48 hours demonstrated that this lectin—like compound was resistant to proteolysis (Figure 4). Bovine serine albumin was hydrolyzed within one hour when incubated with *E*. *kuehniella* midgut proteolytic enzymes (data not shown).

## Discussion

The present study describes the insecticidal and physiological properties of labramin, a lectin—like protein isolated from *L*. *bojeri* seeds. *Ephestia kuehniella* is a polyphagous pest that feeds on a wide variety of stored products. The present study describes the insecticidal properties of labramin, a lectin— like protein isolated from *L*. *bojeri* against *E*. *kuehniella*.

Various plant lectins have shown entomotoxic effects when fed to insects from Coleoptera, Hemiptera, and Lepidoptera (Carlini and Grossi-de-Sá 2002; Lam and Ng 2011). In general, previous studies have tested toxicity by incorporating lectins into artificial diets at concentrations ranging from 1 to 50 ng/g of diet, or from 5 to 1500 µg/mL diet to deliver these proteins to chewing and sucking insects, respectively (Vasconcelos and Oliveira 2004). Purified labramin caused a significant decrease in the weight and survival of the *A*. *kuehniella* fourth—instar larvae, when incorporated into an artificial diet at a level of 1%. The diet containing 0.25% labramin caused a 32% increase in mortality and a 25% reduction in the average weight of the fourth—instar larvae (Figure 1). Generally, addition of 0.25% labramin to the diet prolonged the larval and pupal developmental periods (total of 16 days) and reduced the percentage of emerging adults (mortality ∼57%) compared to the control (Table 1). As such, labramin may play an important role in the control and reduction of the population of this pest.

It has been shown that lectins displaying similar saccharide specificity may have very different effects on closely—related insects (Gatehouse et al. 1995). The LC_50_ of *Koelreuteria paniculata* seed lectin was 0.65%, and the ED_50_ was 0.20% when fed to *E*. *kuehniella* larvae (Macedo et al. 2003). However, for these same insect larvae, *Bauhinia monandra* leaf lectin at a concentration of up to 1% did not significantly decrease survival, but produced a decrease of 40% in larval weight (Macedo et al. 2006).

The larvae reared on the labramin–containing diet consumed less and produced less feces than the control group during the fourth instar (Figure 2A). When the consumption of diet was assessed as a ratio of body weight, no significant difference between the control and the 0.25% labramin treatment was observed (Figure 2B). Thus, there was no evidence that labramin exerts a feeding deterrent effect. Similar results were reported for *Lacanobia oleracea* when fed with Galanthus nivalis agglutin (Fitches et al. 1997), as also observed for the soybean pest *A*. *gemmatalis* (Macedo et al. 2010). The reduction in larval weight, despite the increase in consumption for LbAE—fed larvae, suggests that LbAE inhibits nutrient uptake in *A*. *gemmatalis* (Eisemann et al. 1994).

Therefore, the diet containing 0.25% labramin was less appropriate for *E*. *kuehniella* larval growth, since labramin—fed larvae presented higher metabolic cost indices than control larvae (Table 2). As such, labramin apparently did not affect the larval feeding pattern, as also observed for the soybean pest *A*. *gemmatalis* (Macedo et al. 2010). The reduction in larval weight, despite the increase in consumption by labramin—fed larvae, suggests that labramin inhibits nutrient uptake in *A*. *gemmatalis* (Eisemann et al. 1994). ECI is an overall measurement of an insect's ability to utilize the food that it ingests for growth. A decrease in ECI indicates that more food is being metabolized for energy and less is being converted into body substance (i.e., growth). The proportion of digested food that is actually transformed into net insect biomass is denoted by ECD, the efficiency of conversion of digested food. ECD also decreases as the proportion of digested food metabolized for energy increases (Farrar et al. 1989; Weeler and Isman 2001). In this study, ECI and ECD values decreased by about 27% and 26%, respectively, indicating that labramin exhibits some toxicity and/or antinutritional properties in *E*. *kuehniella* larvae. A greater approximate digestibility value would help to meet the increased demand for nutrients and compensate for the deficiency in foodstuff conversion (reduction in ECI and ECD), perhaps by diverting energy from biomass production into detoxification (Table 2). Coelho et al. (2007) observed similar nutritional indices in *E*. *kuehniella* larvae when they studied an insecticidal lectin from *Annona coriacea* seeds. However, the precise mechanism for the action of lectins in insects is still unknown (Murdock and Shade 2002). A prerequisite for toxicity is that the lectin should be able to survive the proteolytic environment of the insect midgut. Depending on their resistance to gut proteolysis and on their specificity for carbohydrate receptors, lectins may bind to different parts of the intestine to cause various changes in cellular function and morphology (Puztai et al. 1990; Harper et al. 1998; Macedo et al. 2004b). The incubation of labramin with proteases of *E*. *kuehniella* midgut homogenates showed that labramin is resistant to hydrolysis by *E*. *kuehniella* enzymes (Figure 4), and does not demonstrate any inhibition in trypsin activities up to a concentration of 100 µg (data not shown).

To establish whether labramin acts by disrupting the digestive capacity of the larval midgut of *E*. *kuehniella*, here we reported the effects of 0.25% labramin on an enzyme involved in protein digestion, the soluble endoprotease trypsin. Trypsin—like proteases are major digestive enzymes in lepidopteran larvae (Terra et al. 1996; Srinivasan et al. 2006). Labramin stimulated an increase in the trypsin—like activities in the midgut by approximately 19% (Figure 3). Since labramin is known to be resistant to proteolysis, the elevated gut protein levels observed in the feeding assay may partially reflect an accumulation of lectin—like protein bound to larval tissues, which in turn could lead to the induction of trypsin—like activity. Fitches et al. (1998) showed that midgut trypsin secretion can be stimulated by *Canavalia ensiformes* lectin in *L*. *oleracea* larvae.

The peritrophic membrane (PM) is an essential structure for the normal physiology of the insect midgut. It has been suggested that crucial selective pressure during the development of the PM is necessary to avoid the evacuation of digestive enzymes with the feces via the organization of the gut lumen into the ecto— and endo—peritrophic spaces (Terra 2001). The PM consists of proteins, proteoglycans, and chitin (containing Nacetylglucosamine residues) (Wang and Granados 2001). In this study, labramin binding may have affected enzyme—regulatory mechanisms as a consequence of perturbation of the peritrophic membrane environment. Part of this labramin binding probably represents interactions with chitinous structures present in the insect's membranes, since this lectin–like protein bound to a chitin column (Macedo et al. 2004a). Elevated levels of trypsin activity in fecal material were also observed; lectin—like fed insects had ∼23% higher activity than the controls. A change in the membrane environment and consequent disruption of enzyme recycling mechanisms may provide an alternative explanation for the observed increases in the tryptic activity of fecal extracts collected from 0.25% labramin—fed larvae. Recently, a similar effect was verified for *E*. *kuehniella* larvae when fed with Pouterin, a lectin—like protein from *Pouteria torta* seeds (Boleti et al. 2009). In conclusion, labramin may be considered a potential tool for crop protection strategies due to its ability to act on the developmental stages of *the E*. *kuehniella*.
